# Self-critical Rumination and Associated Metacognitions as Mediators of the Relationship Between Perfectionism and Self-esteem

**DOI:** 10.1007/s10942-021-00404-4

**Published:** 2021-06-17

**Authors:** Monica Fearn, Claudia Marino, Marcantonio M. Spada, Daniel C. Kolubinski

**Affiliations:** 1 Division of Psychology, School of Applied Sciences, London South Bank University, London, UK; 2 Dipartimento Di Psicologia Dello Sviluppo e Della Socializzazione, Universita’ di Padova, Padova, Italy

**Keywords:** Perfectionism, Self-Esteem, Self-Critical Rumination, Metacognition

## Abstract

Past research has shown that perfectionism, can negatively impact self-esteem. However, the mediating factors that explain this relationship remain unclear. The current study aimed to investigate whether specific cognitive processes, namely, self-critical rumination and associated metacognitions, mediate this relationship. An opportunity sample of 347 participants completed a battery of online questionnaires measuring clinical perfectionism, self-critical rumination, metacognitions about self-critical rumination, self-esteem, and levels of psychological distress. Several hypotheses were tested to examine the associations between the study variables. Following this, a path analysis was used to determine whether the influence of perfectionistic concerns and perfectionistic striving on self-esteem is mediated by positive metacognitions about self-critical rumination, self-critical rumination, and negative metacognitions about self-critical rumination, serially. Positive metacognitions about self-critical rumination, self-critical rumination, and negative metacognitions about self-critical rumination partially mediated the relationship between perfectionistic concerns and self-esteem and fully mediated the relationship between perfectionistic striving and self-esteem. These results point towards possible interventions for those who struggle with low self-esteem due to their perfectionistic tendencies. Further investigations should explore additional factors that help to explain why perfectionism impacts self-esteem levels, whilst also addressing the limitations of this current research.

## Introduction

A few central components arise in most definitions of perfectionism to emphasize that it involves both the setting of personally demanding standards (Hamachek, 1978) and evaluating oneself in accordance to whether these standards are achieved (Frost et al., 1991). Additionally, attention is likely to be focused on perceived failures rather than any successes accomplished (Hollender, 1965). To date, however, it remains unclear what impact of this process might be on one’s overall evaluation of oneself and the mechanisms that might be involved in that impact.

### Perfectionism

Different authors have debated whether perfectionism is a multidimensional (Frost et al., 1990; Hewitt & Flett, 1991) or a unidimensional (Shafran et al., 2002) construct. Clarifying this, factor analytic studies have consistently found evidence for perfectionism having two higher-order dimensions, namely ‘perfectionistic strivings’ and ‘perfectionistic concerns’ (Bieling et al., 2004; Dickie et al., 2012; Frost et al., 1993; Stoeber & Damian, 2014). Perfectionistic strivings capture the aspect of perfectionism that involves setting and pursuing exceedingly high standards for oneself. Whereas, perfectionistic concerns can be characterised by intense self-scrutiny and concern over one’s mistakes, and additionally a preoccupation regarding others’ judgements (Dunkley et al., 2006).

Differentiating between the two dimensions is important as research has shown that perfectionistic concerns have consistently been associated with negative outcomes including low self-esteem, heightened attachment fears, negative affect, and eating disorders (Dunkley et al., 2003; Dunkley et al., 2012; Frost et al., 1993; Pratt et al., 2001; Rice et al., 1998). Whereas, most research has shown perfectionistic strivings to be associated with positive outcomes such as better academic performance and problem solving skills (Blankstein et al., 2008; Flett et al., 1994; Frost et al., 1993). However, this being said, some studies have also found perfectionistic strivings to be associated with maladaptive outcomes, including increased anxiety and stress (Bieling et al., 2004; Dunkley et al., 2003). In summary, perfectionism is considered to be a two-factorial construct with both maladaptive and adaptive consequences (for a review see Stoeber & Otto, 2006).

### Self-esteem

A considerable body of research has investigated the concept of self-esteem, exploring its potential causes, consequences, and correlates (Marx & Winne, 1978). To define simply, self-esteem refers to the extent that an individual likes themselves and believes they are a person of worth (Brown & Marshall, 2006). It is a subjective measure, thus it does not necessarily reflect one’s objective competencies and successes (Orth & Robins, 2019). High self-esteem is characterised by an evaluation of oneself as worthy and likeable (Rosenberg, 1965), whereas low self-esteem is characterised by ambivalent or negative feelings towards the self (Baumeister et al., 1989).

Self-esteem is thought to remain relatively stable throughout the life span (Trzesniewski et al., 2003); however, occasional fluctuations may occur. This is because self-esteem can become contingent on certain domains such as academic ability, athleticism, popularity, etc. (Crocker & Knight, 2005; Rosenberg et al., 1995; Woike & Baumgardner, 1993). Therefore, one may only feel good about oneself by reaching a certain standard of excellence within a given domain (Crocker & Knight, 2005; Park & Crocker, 2013).

There has been a sizeable debate about the role self-esteem plays with respect to important life outcomes. Several authors have come to the conclusion that self-esteem may have limited importance for one’s life circumstances (Baumeister et al., 2003; Crocker & Park, 2004), whereas others have found evidence suggesting high self-esteem to be predictive of greater job satisfaction (Kuster et al., 2013), physical health (Stinson et al., 2008), relationship satisfaction, and emotional well-being (Orth et al., 2012).

Whilst low self-esteem, on its own, is not considered to be a psychological disorder, it often plays a part in numerous psychopathologies including; generalised anxiety disorder (Henning et al., 2007), major depressive disorder (Orth et al., 2009), eating disorders (Gual et al., 2002; Kugu et al., 2006), substance use disorder (Dooley et al., 2005) and schizophrenia (Barrowclough et al., 2003), to name a few. Overall, the evidence suggests self-esteem is likely to have a significant impact on the quality of life for many individuals.

### Perfectionism and Self-esteem

Many authors have posited a link between perfectionism and self-esteem (Blatt, 1995; Hamachek, 1978; Horney, 1991; Sorotzkin, 1985). It is thought that the self-scrutiny and excessive concern that occurs for individuals displaying perfectionistic concerns produces a disparity between the idealised self and actual self. This disparity results in a more global negative view of the self, thus lowering self-esteem (e.g. Blankstein et al., 2008; Dunkley & Grilo, 2007). In support of this, research has illustrated that increased perfectionistic concerns are associated with lower levels of self-esteem (Dunkley et al., 2012; Flett et al., 1991; Preusser et al., 1994; Rice et al., 1998).

Notably, Taylor et al. (2016) found that when individuals exhibit both perfectionistic concerns and perfectionistic strivings, the influence of perfectionistic concerns may diminish any positive effects that perfectionistic strivings would have had on the individual’s self-esteem. For instance, individuals displaying high perfectionistic strivings and low perfectionistic concerns may have the ability to both strive for high standards and cope with mistakes that may occur. Whilst those exhibiting high perfectionistic strivings and perfectionistic concerns may find themselves experiencing conflict between striving for success and being unable to cope with any mistakes made (Hall et al., 1998; Parker, 1997).

### Self-criticism and Self-critical Rumination

Self-criticism can be defined as persistent, negative evaluations about oneself that occur when standards and expectations are not met (Shahar, 2015). High levels of self-criticism are strongly associated with low self-esteem (Dunkley & Grilo, 2007; Grzegorek et al., 2004; Heimpel et al., 2002), depression (Zuroff et al., 1999) and many other forms of psychological distress (Werner et al., 2019). Research has also found self-criticism acts as a partial mediator in the relationship between clinical perfectionism and psychological distress (Dunkley et al., 2006; James et al., 2015). Nevertheless, some authors have pointed out that self-criticism ‘in moderation’ is not always detrimental, but may help individuals prevent future mistakes, allowing for personal improvement (Driscoll, 1989). Yet, if self-criticism does spiral into a ruminative style of thinking, this is deemed to be problematic in terms of one’s psychological health. (Kolubinski et al., 2016, 2019; Smart et al., 2016).

Rumination is a repetitive and maladaptive thinking style regarding one’s distress (Nolen-Hoeksema et al., 2008; Treynor et al., 2003). Hence, self-critical rumination can be defined as a persistent focus of attention on self-critical thoughts. This usually occurs without any attempt at problem-solving or altering one’s circumstances (Smart et al., 2016). Research exploring self-critical rumination is still in its infancy. Nevertheless, it has been found that self-critical rumination is likely to influence the relationship between self-criticism and psychological distress (Kolubinski et al., 2017; Moreira & Canavarro, 2018). Moreover, self-critical rumination has been found to be a significant predictor of self-esteem, even when controlling for age, levels of self-criticism, stress, anxiety, and depression (Kolubinski et al., 2019).

### Metacognitions about Self-critical Rumination

Information that an individual holds regarding their own internal states and cognitions is defined as ‘metacognition’ (Wells, 2000). Over the past 30 years, there has been a large increase in the literature investigating the role of metacognition in psychological disorders (Wells, 2013). A model proposed by Wells and Matthews (1996), known as the Self-Regulatory Executive Function (S-REF) model, attempts to explain how metacognitions (beliefs about one’s own thinking and how to control it) are involved in the development and maintenance of psychopathology. The model illustrates that psychopathology occurs due to a thinking style called the ‘Cognitive Attentional Syndrome’ (CAS), which consists of excessive worry, rumination, unhelpful and backfiring coping strategies, and attentional biases, such as self-focused attention and attention focused on threats. Two forms of metacognitions—positive and negative, are theorised to be responsible in activating and maintaining the CAS (Wells, 2013). Positive metacognitions are those concerned with the usefulness of engaging in the CAS as a way of improving one’s performance and motivation (e.g., ‘worrying will help me to avoid future problems’), whereas negative metacognitions involve beliefs that engaging in the CAS is both out of one’s control and harmful (e.g., ‘I can’t control my thoughts’; Wells, 2009). Therefore, holding positive metacognitions about the CAS will activate this thinking style, leading to increased rumination and reduced problem solving. If one also holds negative metacognitions, the CAS will remain activated and unabated, fulling greater psychological distress (Wells & Matthews, 1996).

The S-REF model has aided the understanding of processes involved in various forms of distress. Some examples include; depressive rumination (Papageorgiou & Wells, 2001a, 2001b), problem gambling (Spada et al., 2014), problem drinking (Caselli & Spada, 2013; Spada & Wells, 2006) and anger (Simpson & Papageorgiou, 2003). Similarly, the S-REF model can help explain the metacognitive processes responsible for the amplification and maintenance of self-critical rumination (Kolubinski et al., 2016), as research has found both positive and negative metacognitions to be predictive of self-critical rumination, even when levels of self-esteem, self-criticism and affect are controlled for (Kolubinski et al., 2017).

A recent study conducted by Kolubinski and colleagues (2019) explored the impact that self-critical rumination, and associated metacognitions, have on self-esteem. The model suggests that self-critical thoughts, when combined with positive metacognitions about self-critical rumination, would lead to the activation of self-critical rumination. If one simultaneously holds negative metacognitions about self-critical rumination, which is strongly associated with self-critical rumination, it will work to maintain that process of thinking. Prolonged exposure to this thinking style is likely to lower one’s self esteem. Their findings supported this model, thus showing self-critical rumination and the associated metacognitions are likely to play a crucial role in predicting low self-esteem.

### Study Objectives and Hypotheses

The aim of the current study was to investigate whether self-critical rumination, and associated positive and negative metacognitions, mediate the relationship between perfectionism and self-esteem. Previous literature has shown that perfectionistic concerns are associated with lower self-esteem (e.g. Dunkley et al., 2012; Flett et al., 1991; Rice et al., 1998), higher levels of self-criticism, and increased ruminative tendencies (James et al., 2015; O’Connor et al., 2007). Research has also found that both self-critical rumination and metacognitions about self-critical rumination play a significant role in predicting lower self-esteem (Kolubinski et al., 2019). What is less clear, however, is whether the self-critical rumination that occurs due to the associated positive and negative metacognitions mediates the relationship between perfectionistic concerns and self-esteem.

This research proposes that an individual who embodies a high level of perfectionistic concerns or perfectionistic striving is likely to hold positive metacognitions about self-critical rumination, which will lead to ruminating about self-critical thoughts. If the individual also holds negative metacognitions about self-critical rumination, this will serve to maintain self-critical rumination, impacting their self-esteem. This prediction is in accordance with the S-REF model (Wells & Matthews, 1996).

In order to assess this, several hypotheses were tested. Firstly, it was hypothesised that self-esteem would be negatively correlated with perfectionistic concerns, perfectionistic striving, self-critical rumination, and both positive and negative metacognitions about self-critical rumination. Secondly, it was hypothesised that the other variables would be positively correlated with each other. Lastly, it was hypothesised that perfectionistic concerns and perfectionistic striving would indirectly influence self-esteem through the effect on positive metacognitions about self-critical rumination, self-critical rumination, and negative metacognitions about self-critical rumination, serially, and when controlling for levels of psychological distress.

## Method

### Participants

The sample consisted of 347 individuals ranging in age from 18 to 95 years old (*M* = 41.58, *SD* = 16.35, 31 chose not to respond). Recruitment of participants was carried out via opportunity sampling by advertising the study online using both social media platforms and the research participation scheme at London South Bank University. Individuals taking part in the study were required to be at least 18 years of age and be able to understand and communicate using English. A power analysis determined that a sample size of 103 participants was required in order to detect a medium effect size with a power of 0.80.

### Self-report Measures

#### Clinical Perfectionism

The Clinical Perfectionism Questionnaire (CPQ; Shafran et al., 2002) measures both perfectionistic strivings (CPQ-PS) and perfectionistic concerns (CPQ-PC). This questionnaire uses a 4-point Likert scale ranging from 1 (‘not at all’) to 4 (‘all of the time’), with item 2 being reversed scored. Overall, higher scores indicate higher levels of clinical perfectionism. Participants are asked to report how they have felt ‘over the past month’ with questions such as, “Have you pushed yourself really hard to meet your goals?”. The CPQ consists of 12 items, however, in the current study items 7 and 8 were removed. Item 7 was found to have a high cross-loading so it was not clear which factor this item measured and item 8 was found to be problematic when measuring clinical perfectionism in a non-clinical sample (Dickie et al., 2012). The resulting 10 items measure the two distinct dimensions of clinical perfectionism: (1) perfectionistic strivings, displaying good internal consistency (α = 0.78); and, (2) perfectionistic concerns, showing satisfactory internal consistency (α = 0.68).

#### Self-critical Rumination

The Self-critical Rumination Scale (SCRS; Smart et al., 2016) measures ruminative processes related to self-critical thoughts. This measure includes 10 items, each using a 4-point Likert scale ranging from 1 (‘not at all’) to 4 (‘very much’). Higher scores on this questionnaire signify increased levels of self-critical rumination (e.g., “My attention is often focused on aspects of myself that I’m ashamed of”. Three of the items (#3, 4, 7), however, represent metacognitions about self-critical rumination (e.g., “Sometimes it is hard for me to shut off critical thoughts about myself”), so they were removed for the purpose of this study with a view to better distinguish between self-critical rumination and the metacognitions related to it. The resulting 7 questions of the modified version of the SCRS (SCRS-M) still maintained excellent reliability (α = 0.91) and correlated very strongly with the original 10-item version (r = 0.98, *p* < 0.001).

#### Metacognitions about Self-critical Rumination

The Metacognitions about Self-critical Rumination questionnaire (MSCRQ; Kolubinski et al., 2017) measures both positive (MSCRQ-P) and negative (MSCRQ-N) metacognitions associated with self-critical rumination. There are 10 items, each using a 4-point Likert scale from 1 (‘do not agree’) to 4 (‘agree very much’). Items 2, 3, 5 and 7 assess the presence of MSCRQ-P (e.g., “I motivate myself to try harder by dwelling on stupid things I did in the past”). The MSCRQ-P subscale had a satisfactory internal consistency (α = 0.68). Items 1, 4, 6, 8, 9 and 10 assess MSCRQ-N (e.g., “I will get depressed if I don’t stop reviewing my self-critical thoughts”). This subscale showed very good internal consistency (α = 0.81) and correlates very strongly with the SCRS (Kolubinski et al., 2019).

#### Self-esteem

The Rosenburg Self-Esteem Scale (RSES; Rosenberg, 1965) is a widely used measurement of self-esteem. It includes 10 items, each using a 4-point Likert scale ranging from 0 (‘strongly disagree’) to 3 (‘strongly agree’), with items 3, 5, 8, 9 and 10 being reversed in valence. A higher score indicates higher levels of self-esteem. Participants are asked to report how much they agree with each statement (e.g., “I feel that I’m a person of worth, at least on an equal plane with others.”) This measure displayed excellent internal consistency (α = 0.90).

#### Psychological Distress

The short form of the Depression Anxiety Stress Scale (DASS-21; Antony et al., 1998) measures general psychological distress, including symptoms of depression, hyperarousal and tension. It consists of 21 items, each using a 4-point Likert scale ranging from 0 (‘Did not apply to me at all’) to 3 (‘Applied to me most of the time’). A higher score indicates higher levels of psychological distress. The questionnaire asked participants to report how much each statement (e.g., “I found it hard to wind down”) applied to them over the past week. The DASS-21 displayed excellent internal consistency (α = 0.92).

### Procedure

Ethical approval for this study was granted by the Division of Psychology Research Ethics Committee at London South Bank University. Participants were recruited via various social media platforms (Facebook, Instagram, Email, and WhatsApp) by sharing a hyperlink to the study website. Those who took part were encouraged to forward on the hyperlink to their own contacts in attempt to gather a wide range of participants. Additionally, the research participation scheme at London South Bank University was used to recruit undergraduate psychology students.

The hyperlink directed participants to the study website where they were presented with a participant information sheet explaining the purpose of the research, what it will entail, and details about anonymisation and confidentiality of responses. If consenting to take part, participants were presented with the following self-report measures to complete in order: DASS-21, CPQ, SCRS, MSCRQ, and lastly the RSES. Following completion of the questionnaires, the participants were thanked and debriefed.

### Data Analysis

**Fig. 1:**
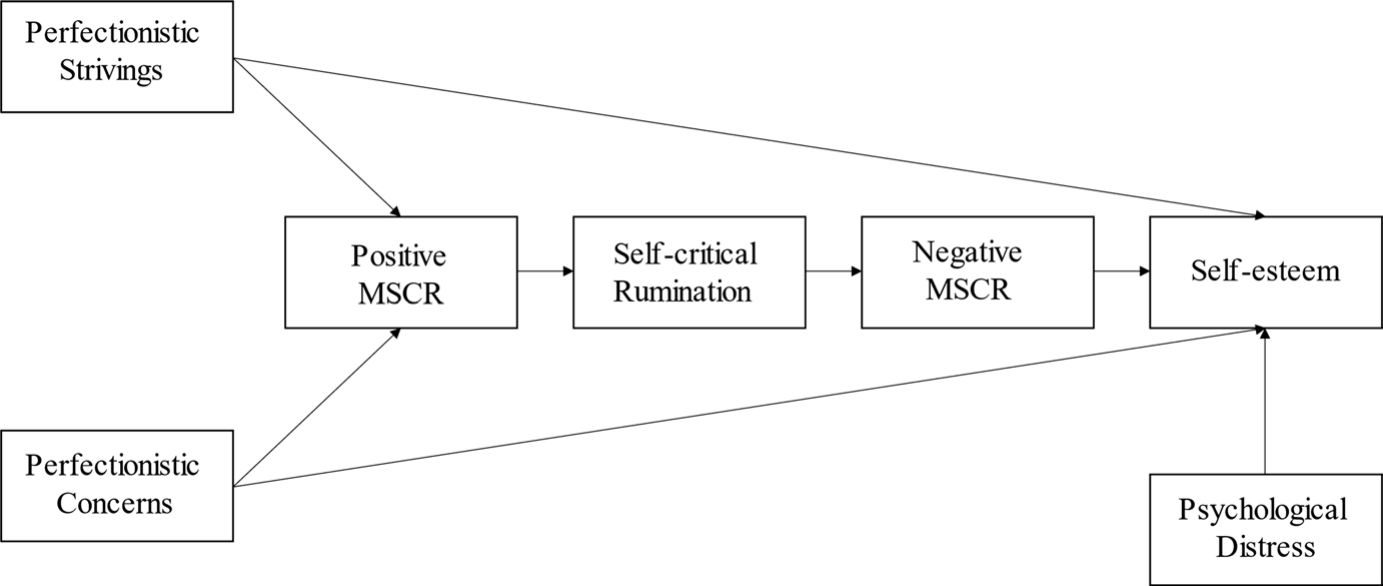
Proposed theoretical model (MSCR = Metacognitions about Self-critical Rumination)

SPSS (version 26; IBM Corp., 2019) was used to conduct correlation analyses to test the associations between all variables in the study. A path analysis approach was then applied to test the association among study variables, using R (R Development Core, 2017) and single observed score for each construct. The two dimensions of perfectionism (CPQ-PS and CPQ-PC) were included as independent variables; positive metacognitions about self-critical rumination (MSCRQ-P), self-critical rumination (SCRS) and negative metacognitions about self-critical rumination (MSCRQ-N) as mediators; and self-esteem (RSES) as dependent variable. Psychological distress (DASS) was included in the model as covariate of RSES (Fig. 1). The age variable contained 31 missing values and was not included in the model in order to retain as many participants as possible in the path analysis. The robust maximum likelihood estimator (MLR; Satorra & Bentler, 1994), suitable for non-normally distributed variables, was used. Indirect paths from the independent variables to the dependent variable via mediators were tested using the Sobel tests for mediation (Baron & Kenny, 1986; Hayes, 2013). To evaluate the model fit, the explained variance of each endogenous variable (R2) and the total coefficient of determination (TCD; Jöreskog & Sörbom, 1996) were considered.

## Results

### Distribution of Data and Bivariate Correlations

A series of Shapiro–Wilk normality tests showed that all variables were non-normally distributed at the *p* < 0.05 level. Correlation analyses, using Spearman’s Rho, were conducted to test the associations between all variables in the study. These variables included participant age, perfectionistic concerns (CPQ-PC), perfectionistic strivings (CPQ-PS), self-critical rumination (SCRS), positive metacognitions about self-critical rumination (MSCRQ-P), negative metacognitions about self-critical rumination (MSCRQ-N), self-esteem (RSES) and psychological distress (DASS). The means, standard deviations, and interquartile ranges are displayed in Table 1.

**Table 1:** Means, standard deviations, medians, and interquartile ranges

		Mean	S.D	Median	Interquartile range
1	Age	41.51	16.37	42.00	29
2	CPQ-PC	5.16	2.24	5.00	3.00
3	CPQ-PS	8.05	3.45	8.00	4.00
4	SCRS-M	9.47	5.59	9.00	9.00
5	MSCRQ-P	3.59	2.36	3.00	3.00
6	MSCRQ-N	4.72	3.72	4.00	5.00
7	RSES	18.78	5.30	19.00	7.00
8	DASS-21	15.29	9.44	14.00	13.00

*n* 347; *p < .05. **p < .001. CPQ-PC = Clinical Perfectionism Questionnaire—Perfectionistic concerns; CPQ-PS = Clinical Perfectionism Questionnaire—Perfectionistic strivings; SCRS-M = Modified version of the Self-Critical Rumination Scale; MSCRQ-P = Metacognitions about Self-Critical Rumination Scale—Positive; MSCRQ-N = Metacognitions about Self-Critical Rumination Scale—Negative; RSES = Rosenberg Self-Esteem Scale; DASS-21 = Depression, Anxiety Stress Scale-21

The analysis showed all study variables were significantly correlated with each other at the p < 0.001 level. As expected, significant negative correlations were found between RSES and CPQ-PC (r_s_ = -0.66, p < 0.001), CPQ-PS (r_s_ = -0.32, p < 0.001), SCRS (r_s_ = -0.73, p < 0.001), MSCRQ-P (r_s_ = -0.46, p < 0.001) and MSCRQ-N (r_s_ = -0.72, p < 0.001). Additionally, significant positive correlations were found between the study variables CPQ-PC, SCRS, MSCRQ-P and MSCRQ-N (See Table 2).

**Table 2:** Bivariate correlations

		1	2	3	4	5	6	7
1	Age	–						
2	CPQ-PC	− .35**	–					
3	CPQ-PS	− .30**	.50**	–				
4	SCRS-M	− .38**	.70**	.46**	–			
5	MSCRQ-P	− .30**	.40**	.44**	.59**	–		
6	MSCRQ-N	− .28**	.62**	.40**	.75**	.57**	–	
7	RSES	.24**	− .66**	− .32**	− .73**	− .50**	− .72**	–
8	DASS-21	− .28**	.60**	.38**	.64**	.40**	.55**	− .56**

*n* 347; *p < .05. **p < .001. CPQ-PC = Clinical Perfectionism Questionnaire—Perfectionistic concerns; CPQ-PS = Clinical Perfectionism Questionnaire—Perfectionistic strivings; SCRS-M = Modified version of the Self-Critical Rumination Scale; MSCRQ-P = Metacognitions about Self-Critical Rumination Scale—Positive; MSCRQ-N = Metacognitions about Self-Critical Rumination Scale—Negative; RSES = Rosenberg Self-Esteem Scale; DASS-21 = Depression, Anxiety Stress Scale-21

### Assessing Multicollinearity

Due to the high correlations listed above, the Variance Inflation Factors (VIF) were calculated for all predictor variables. No VIF exceeded the cut-off of 10 (Max = 3.17; Kutner, Nachtsheim, & Neter, 2004; Sheather, 2009). The original 10-item SCRS, however, resulted in VIFs between 5.5 and 5.8 when all but two predictor variables were used as the criterion (MSCRQ-N and DEQ-SC6), which questions the overlap in variance between self-critical rumination, its negative metacognitions and self-criticism.

### Path Analysis

**Fig. 2:**
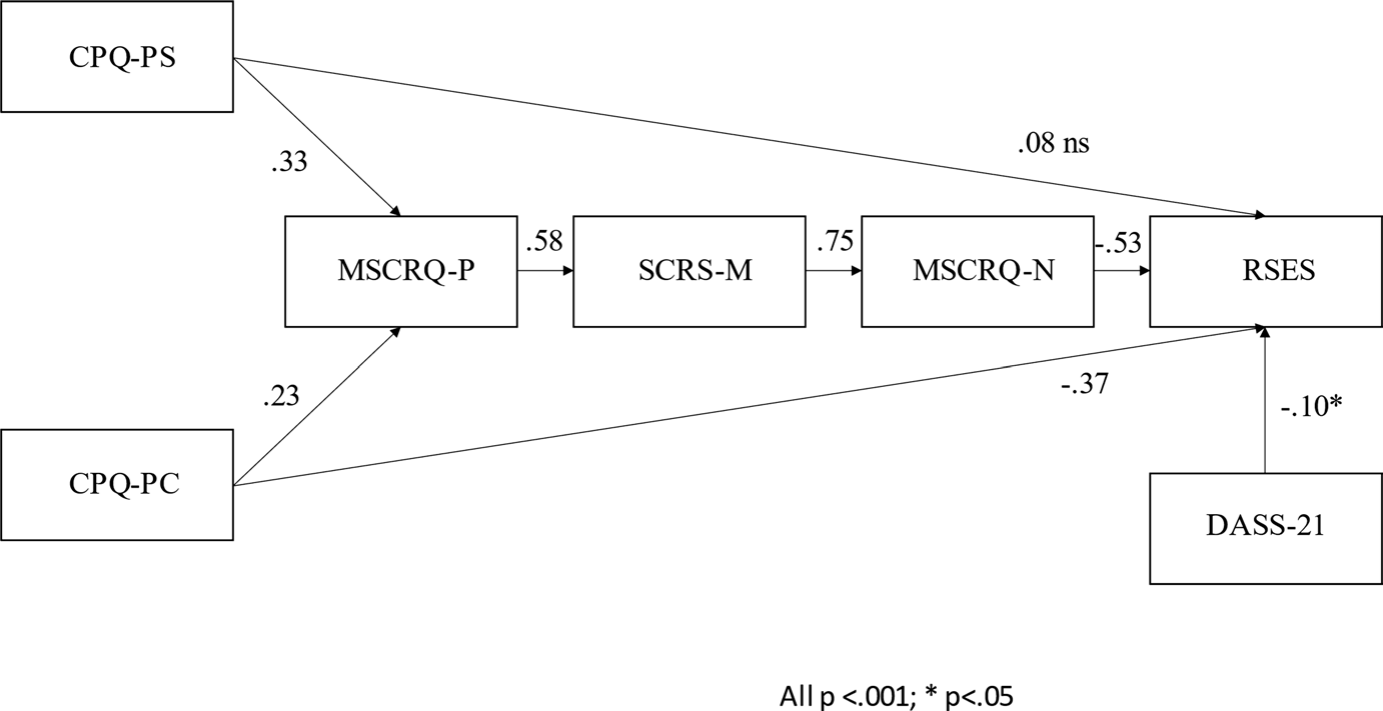
Results of the path analytical model. Notes: *n* = 316; CPQ-PS = Clinical Perfectionism Questionnaire—Perfectionistic strivings; CPQ-PC = Clinical Perfectionism Questionnaire—Perfectionistic concerns; MSCRQ-P = Metacognitions about Self-Critical Rumination Scale—Positive; SCRS-M = Modified version of the Self-Critical Rumination Scale; MSCRQ-N = Metacognitions about Self-Critical Rumination Scale—Negative; RSES = Rosenberg Self-Esteem Scale; DASS-21 = Depression, Anxiety Stress Scale-21; All *p* < .001; *p < .05

As shown in the Fig. 2, results of the path analyses revealed that all path coefficients were significant at the *p* < 0.001 level, with the exception of the association between DASS and RSES (β = -0.10, *p* < 0.05) and the link between CPQ-PS and RSES that was not significant (β = 0.08, *p* = 0.09). Conversely the other dimension of perfectionism (CPQ-PC) was directly associated with RSES.

Both CPQ-PS and CPQ-PC were positively associated with MSCRQ-P, which, in turn, was positively associated with SCRS. Moreover, SCRS was positively and strongly associated with MSCRQ-N, which, in turn, was negatively associated with the outcome variable (RSES).

With regards to indirect relationships, results of the Sobel test supported the mediating role of the three mediators between the two dimensions of perfectionism and self-esteem: namely, the indirect link between CPQ-PS and RSES via three mediators (MSCRQ-P SCRS MSCRQ-N) (β = -0.075, SE = 0.023, z = -4.487, *p* < 0.001), and the indirect link between CPQ-PC and RSES via three mediators (β = -0.037, SE = 0.047, z = -2.803, *p* = 0.005).

With regards to model fit, the model accounted for 51% of the variance for the outcome variable (RSES), 56% of the variance for one mediator (i.e. MSCRQ-N) variable. Lower variance was observed for the other mediators (e.g., 34% for SCRS and 24% for MSCRQ-P). Finally, the total amount of variance explained by the model (Total Coefficient of Determination, TCD = 0.41) indicated a good fit to the observed data. Indeed, this TCD corresponds to a correlation of r = 0.64, which can be considered a medium to large effect size (Cohen, 1988).

## Discussion

### Addressing the Aims of the Study

The primary aim of this study was to investigate whether the influence of perfectionism, as distinguished by perfectionistic concerns and perfectionistic striving, on self-esteem was mediated, serially, by positive metacognitions about self-critical rumination, self-critical rumination, and negative metacognitions about self-critical rumination in accordance with the S-REF model (Wells & Matthews, 1996).

Prior to investigating this mediation model, evidence was found supporting all other hypotheses stated at the outset of the research. Firstly, self-esteem was negatively correlated in the moderate to high range with perfectionistic concerns, perfectionistic striving, self-critical rumination, positive metacognitions about self-critical rumination and negative metacognitions about self-critical rumination. Secondly, perfectionistic concerns, perfectionistic striving, self-critical rumination, and metacognitions about self-critical rumination (positive and negative) were found to be positively correlated with one another. The majority of these associations have been apparent in previous literature (e.g. Dunkley et al., 2012; James et al., 2015; Kolubinski et al., 2016, 2017, 2019; Rice et al., 1998). However, until now evidence that perfectionism and self-critical rumination has not yet emerged in the literature. This finding enriches earlier research, as previous studies have found positive associations between perfectionistic concerns and other forms of rumination, such as ‘brooding’ rumination (Egan et al., 2014; O’Connor et al., 2007) and depressive rumination (Flett et al., 2002), but not self-critical rumination, which despite having similarities to other ruminative processes, is considered to be its own distinct construct (Smart et al., 2016).

In further support of the hypotheses, the path analysis indicated that both perfectionistic concerns and perfectionistic striving indirectly influenced self-esteem levels through their effect on positive metacognitions about self-critical rumination, self-critical rumination, and negative metacognitions about self-critical rumination, in a sequential manner. In the case of perfectionistic concerns, which includes increased self-criticism, this implies that the presence of positive metacognitions about self-critical rumination would activate self-critical rumination. If negative metacognitions about self-critical rumination are also present, this would further increase the difficulty in shifting attention away from self-critical thoughts as attempts at interrupting self-critical rumination would be discontinued or not initiated in the first place. Over time, as self-critical rumination becomes perseverative, adverse effects on self-esteem should ensue. These findings extend the existing literature, aligning themselves with the metacognitive model of self-esteem (Kolubinski et al., 2019), which is grounded in Wells and Matthews’ (1996) S-REF model.

In support of the present findings, prior research has shown that those high in perfectionistic concerns tend to utilise maladaptive coping mechanisms, such as rumination, rather than active problem-solving, to deal with setbacks (Mouratidis & Michou, 2011; Park et al., 2010). Rumination is an emotion-focused coping mechanism, which is utilised in the attempt to process and modify internal negative events, whereas problem-focused coping involves guiding action with the intention to alter one’s external reality (Folkman, 2013; Wells & Matthews, 1996). Coping via ruminative thinking, thus avoiding active problem-solving, means that self-critical cognitions go unchallenged. Consequently, the acquisition of new and effective skills are obstructed (Wells, 2000). This has been shown using experimental studies that have induced rumination in individuals experiencing distress. For example, Lyubomirsky and Nolen-Hoeksema (1995) found rumination to interfere with inter-personal problem solving and to have an adverse effect on mood. By virtue of the literature, the perfectionists’ inclination to use rumination as a way of coping, is unhelpful and likely prolongs psychological distress.

Kolubinski et al. (2019), upholding the present findings, showed that prolonged exposure to self-critical rumination, linked to one’s metacognitions, has the potential to negatively impact self-esteem. This is important to understand as lowered self-esteem may have further adverse effects on many important life outcomes (Orth et al., 2012; Trzesniewski et al., 2006). Two opposing theories are often highlighted in the literature, namely the ‘scar model’ and the ‘vulnerability model’ of self-esteem. The scar model states that negative affect impacts self-esteem levels, whereas the vulnerability model suggests low self-esteem leads to the development of distress (Orth et al., 2009, 2016; Shahar & Davidson, 2003; Shahar & Henrich, 2010). Both theories have been supported in the literature, and this relationship may be reciprocal. A greater evidence-base does favour, however, the vulnerability model (Ormel et al., 2004; Orth et al., 2009; Shahar & Davidson, 2003; Sowislo & Orth, 2013), signifying that low self-esteem is a strong risk factor for the development of psychological distress. This is crucial to understand, as interventions aimed at restructuring negative self-evaluation could prevent the development of further psychological distress.

Unlike perfectionistic striving, the present research also uncovered that perfectionistic concerns directly influenced self-esteem levels, independently of the other variables in the study. This suggests that the cognitive processes proposed to explain the relationship only partially mediate the effect of perfectionistic concerns on self-esteem levels. Therefore, it is likely there are more factors that will need to be understood to gain a full picture. One possible addition to the proposed pathways could be one’s inter-personal relationships. Rumination has been thought to impair social relationships (Young & Nolen-Hoeksema, 2001), and according to the Sociometer theory (Leary et al., 1995), self-esteem is dependent on the degree to which one is included by others, whereby rejection from one’s peers will lead to lower self-esteem. Thus, if the individual starts to lose peer support due to excessive rumination, this may consequently impact their self-esteem levels. Future research may therefore wish to explore inter-personal relationships as an additional factor to the proposed pathway in this study. Other additional factors that are worth exploring include: parental pressure, schooling environment, socio-economic status, social inequalities, etc.

### Clinical Implications

Results obtained from the present research have fostered a greater theoretical understanding of the pathways involved in explaining how perfectionistic concerns can lead to low self-esteem. These findings point towards therapeutic practices that are likely to benefit individuals by decreasing perfectionistic tendencies and rumination as a way of improving global self-evaluation.

A suitable intervention to consider is Metacognitive Therapy (MCT), which is theoretically grounded in the S-REF model (Wells & Matthews, 1996). This therapy promotes recovery by modifying one’s metacognitions that work to maintain the Cognitive Attentional Syndrome (CAS). The CAS is a maladaptive constellation of coping strategies including, but not limited to, thought suppression, rumination, excessive worry, threat monitoring and dwelling on the past (Wells, 2013). By identifying the metacognitions that maintain the CAS, the individual can learn to alter these and acquire novel ways in which to respond to negative intrusive thoughts. Some of the practices used in MCT include the attention training technique (Fergus & Bardeen, 2016; Fergus et al., 2014; Knowles et al., 2016), detached mindfulness (Gkika & Wells, 2015; Wells, 2005), worry mismatch and rumination postponement (Wells, 2013). These all work towards increasing the flexibility of how one responds when experiencing unhelpful cognitions. Evidence has shown promising effects of utilising MCT for psychological distress (Nordahl et al., 2017; Solem et al., 2009; Wells & Colbear, 2012; Wells et al., 2010). Considering both the present and earlier research evidence, it is likely that MCT may provide a promising clinical application for individuals who struggle to separate themselves from their harmful perfectionistic, self-critical thinking.

### Limitations

When interpreting the results of this research, several limitations ought to be considered. First, whilst the sample was diverse with respect to age, the participants’ gender, ethnicity, and psychiatric history was not recorded in this study, therefore it is unknown whether the findings can generalise to all demographic groups or whether any of the participants have been involved in psychological treatment. Second, all data was collected via self-report questionnaires, meaning the results are subject to possible biases such as social desirability and context effects. Third, a cross-sectional design was used, thus posing limits when inferring a causal relationship between the variables. Fourth, it is uncertain that the self-report questionnaires accurately measured the constructs they set out to measure. In particular, the questionnaire items measuring positive metacognitions about self-critical rumination (MSCRQ) and perfectionistic concerns (CPQ) had a slightly lower Cronbach’s alpha scores, suggesting there may be a problem with internal consistency. Additionally, the self-critical rumination scale (SCRS; Smart et al., 2016) includes three items that assess negative metacognitions. These items were not removed for this study, so it may be that the SCRS measured, in part, negative metacognitions rather than purely self-critical rumination. Fifth, opportunity sampling was used to recruit participants, thus it is possible the sample was not representative of the entire population. Sixth, this study did not control for potential confounding variables, such as socio-economic status, education, social relationships, support networks or being in psychological treatment. Lastly, the data was collected during the height of the COVID-19 pandemic, affecting individuals in a variety of ways, including self-reported measures of cognitive-affective states. Future studies should address these limitations.

## Conclusion

The current research makes a substantial theoretical contribution to the understanding of how perfectionistic concerns can impact one’s self-esteem via specific cognitive and metacognitive processes. The findings point towards clinical implications, such as the use of Metacognitive Therapy for individuals who struggle with low self-esteem due to perfectionism. Further investigations could extend this research by exploring other additional factors that help to explain why perfectionistic concerns impact self-esteem, whilst also addressing the limitations of the present study. To conclude, it may be possible to prevent individuals with perfectionistic tendencies from developing low self-esteem by altering the metacognitions that activate and maintain their self-critical rumination.

## Data Availability

Data available on request.
